# Author Correction: Epiblast-derived CX3CR1+ progenitors generate cardiovascular cells during cardiogenesis

**DOI:** 10.1038/s44318-025-00635-6

**Published:** 2025-11-12

**Authors:** Kyuwon Cho, Mark Andrade, Saeed Khodayari, Christine Lee, Seongho Bae, Sangsung Kim, Jin Eyun Kim, Young-Sup Yoon

**Affiliations:** 1 Department of Medicine, Division of Cardiology, Emory University School of Medicine, Atlanta, GA 30322 USA; 2 Severance Biomedical Science Institute, Yonsei University College of Medicine, Seoul, Republic of Korea

## Abstract

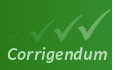

**Correction to:** *The EMBO Journal* (2025) 44:4331–4351. 10.1038/s44318-025-00488-z | Published online 23 June 2025

**The author list is corrected**.

The 3rd author’s name is corrected to Saeed Khodayari.

