# Supplementary material for: Epiblast-derived CX3CR1+ progenitors generate cardiovascular cells during cardiogenesis
Source: EMBO J. 2025 Jun 23;44(15):4331–51. doi: 10.1038/s44318-025-00488-z (PMC12316875; doi:10.1038/s44318-025-00488-z)
Supplement: Supplementary file 2 — Appendix [file 44318_2025_488_MOESM2_ESM.pdf]

Appendix

Epiblast-derived CX3CR1+ progenitors generate cardiovascular cells during cardiogenesis

Kyuwon Cho, Mark Andrade, Khodayari, S. Khodayari, Christine Lee, Seongho Bae, Sangsung Kim, Jin Eyun Kim, Young-sup Yoon*

***Correspondence to:** Young-sup Yoon, M.D., Ph.D., Division of Cardiology, Department of Medicine, Emory University School of Medicine, 1750 Haygood Dr. NE, HSRBII, N248, Atlanta, GA 30322, USA, E-mail: yyoon5@emory.edu, Tel: 404-727-8176

Table of Content:

Appendix Figure S1……………………………………………………………………Page 2-3

Appendix Figure S2……………………………………………………………………Page 4-5

Appendix Figure S3……………………………………………………………………Page 6

Appendix Figure S4……………………………………………………………………Page 7-8

Appendix Figure S5……………………………………………………………………Page 9

Appendix Figure S6……………………………………………………………………Page 10-11

Appendix Figure S7……………………………………………………………………Page 12-13

Appendix Figure S8……………………………………………………………………Page 14-15

Appendix Figure S9……………………………………………………………………Page 16-17

Appendix Figure S10…………………………………………………………………Page 18-19

Appendix Table S1……………………………………………………………………Page 20

Appendix Table S2……………………………………………………………………Page 20

Appendix Table S3……………………………………………………………………Page 20

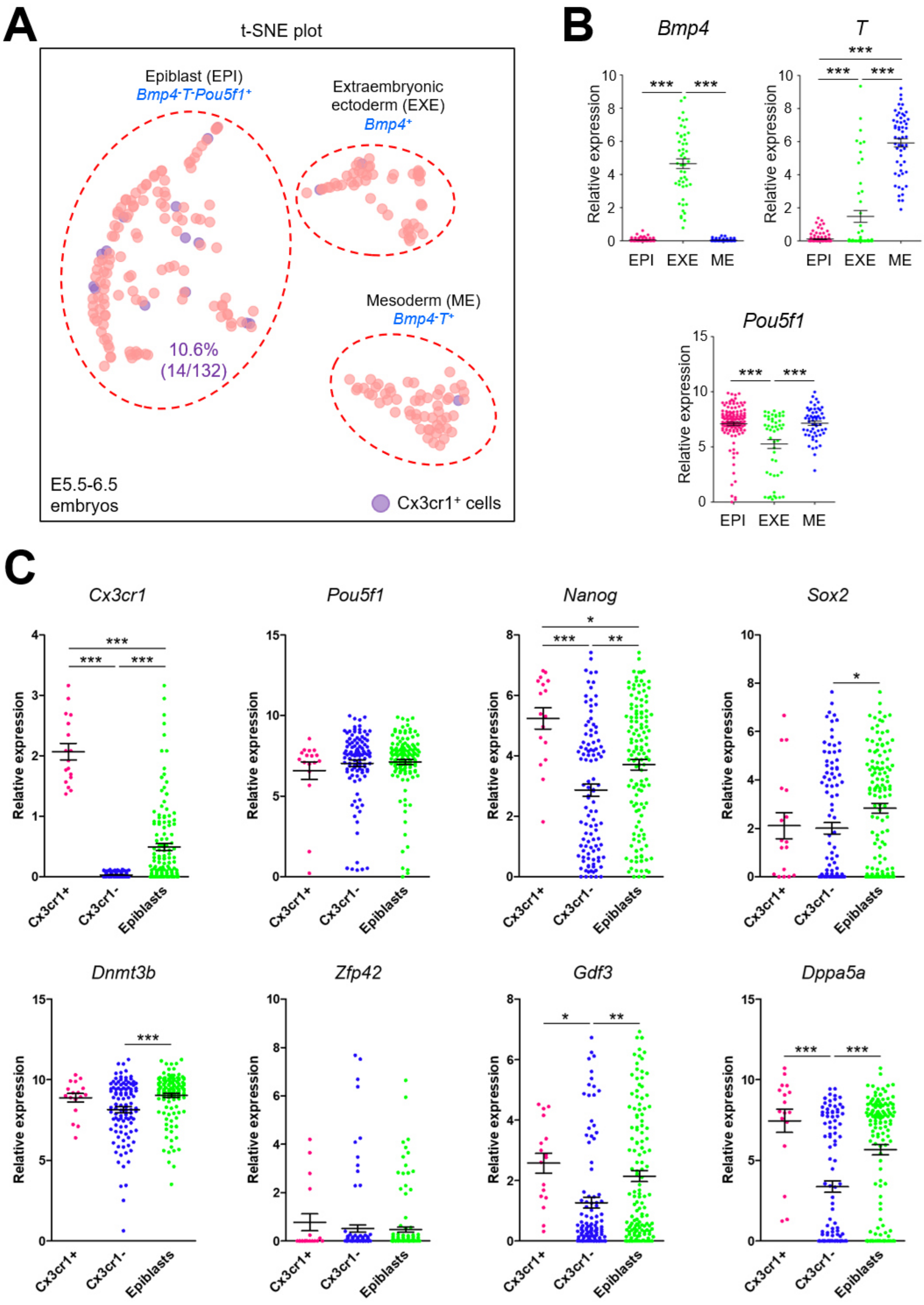

A
t-SNE plot
Epiblast (EPI)
Bmp4⁻T⁻Pou5f1⁺
Extraembryonic ectoderm (EXE)
Bmp4⁺
Mesoderm (ME)
Bmp4⁻T⁺
10.6%
(14/132)
E5.5-6.5 embryos
Cx3cr1⁺ cells
B
Bmp4
T
Pou5f1
Relative expression
EPI
EXE
ME
C
Cx3cr1
Pou5f1
Nanog
Sox2
Dnmt3b
Zfp42
Gdf3
Dppa5a
Relative expression
Cx3cr1+
Cx3cr1-
Epiblasts

Appendix Figure S1. scRNA-seq analysis of *Cx3cr1*$^{+}$ cells in mouse embryos at E5.5-6.5.

A-C. To examine molecular signatures of *Cx3cr1*$^{+}$ cells, we utilized the previously published scRNA-seq data (Wen et al) where embryos were collected at E5.5-6.5 and subjected to scRNA-seq. **A.** t-SNE visualization of 236 mouse embryonic cells at E5.5-6.5. Cell clusters were annotated on the basis of expression pattern of marker genes including *Bmp4, T* and *Pou5f1*. EPI, epiblast; EXE, extraembryonic ectoderm; ME, mesoderm. The numbers inside the EPI population indicate the percentage of *Cx3cr1*$^{+}$ population out of total EPI cells (14 cells out of 132 cells). *Cx3cr1*$^{+}$ cells were defined by the expression of *Cx3cr1* above mean + 1.5SD (standard deviation). **B.** Relative expression of *Bmp4, T,* and *Pou5f1* in each cluster. **C.** Relative expression of pluripotency markers in *Cx3cr1*$^{+}$ cells, *Cx3cr1*$^{-}$ cells and whole epiblasts. *Cx3cr1*$^{+}$ cells were defined by the expression of *Cx3cr1* above mean + 1.5SD and *Cx3cr1*$^{-}$ cells were defined by the expression of *Cx3cr1* below mean - 0.5SD. Error bars: standard error of mean. One-way ANOVA was performed followed by a Tukey HSD test. *P < 0.05, **P < 0.01, ***P < 0.001.

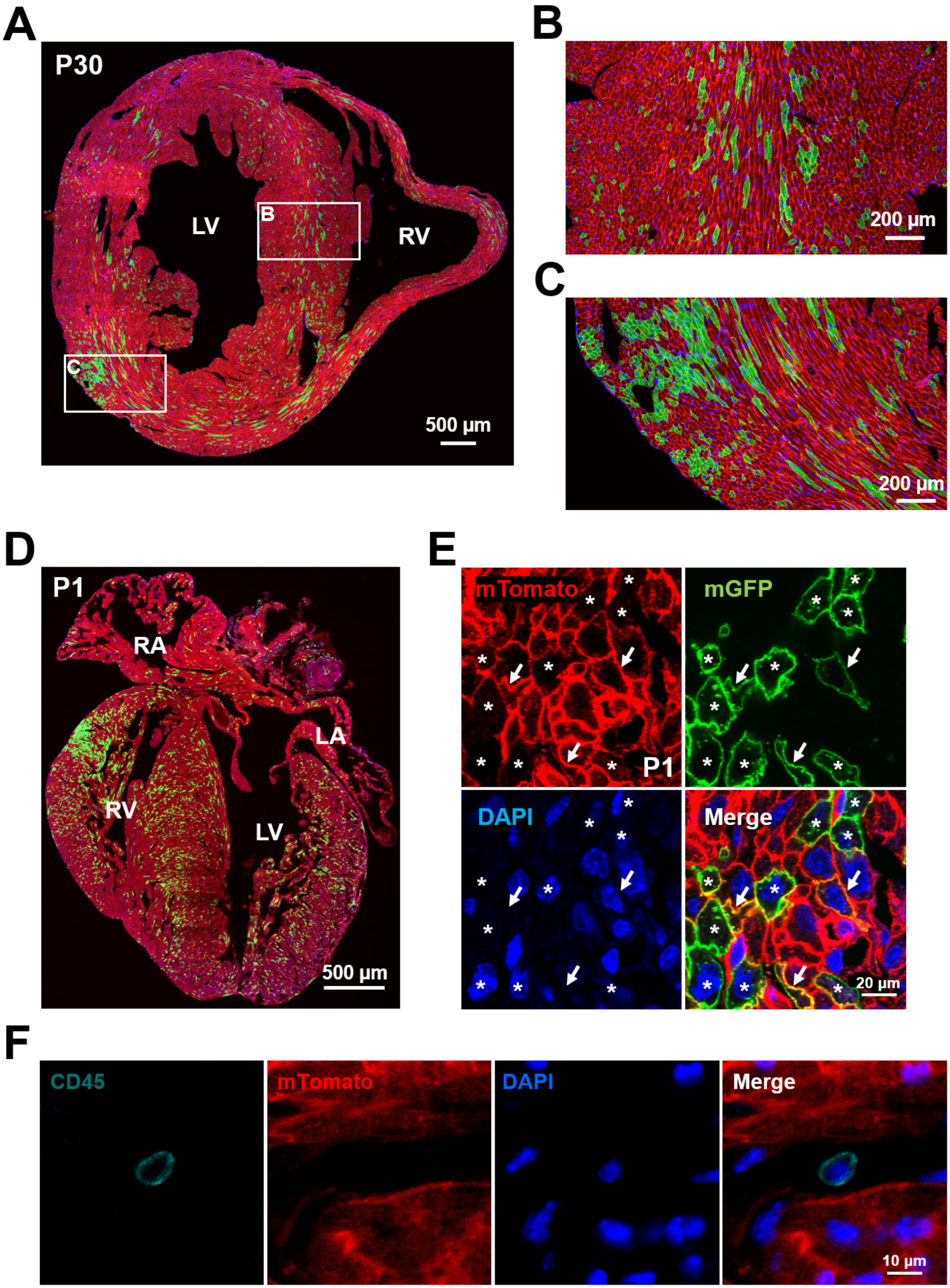
A
P30
LV
RV
B
C
500 µm
B
200 µm
C
200 µm
D
P1
RA
LA
RV
LV
500 µm
E
mTomato
mGFP
P1
DAPI
Merge
20 µm
F
CD45
mTomato
DAPI
Merge
10 µm

Appendix Figure S2. Generation of CMs by CX3CR1$^+$ cells via both *de novo* differentiation and fusion with pre-existing CMs. A. A confocal microscopic image of the whole heart from the *Cx3cr1-cre;R26-mT/mG* young adult mouse (P30) in a two-chamber view. **B-C.** Magnified confocal microscopic images of boxed areas in panel A. **D.** A confocal microscopic image of the whole heart from the *Cx3cr1-cre;R26-mT/mG* neonatal mouse (P1) in a four-chamber view. LA, left atrium; LV, left ventricle; RA, right atrium; RV, right ventricle. **E.** Magnified confocal microscopic images of the neonatal heart with individual color panels. Arrows indicate mTomato$^+$mGFP$^+$ cells. Asterisks indicate only mTomato-positive cells. DAPI (blue). **F.** A representative confocal microscopic image of the *Cx3cr1-cre;R26-mT/mG* young adult heart (P30) immunostained for CD45. DAPI (blue).

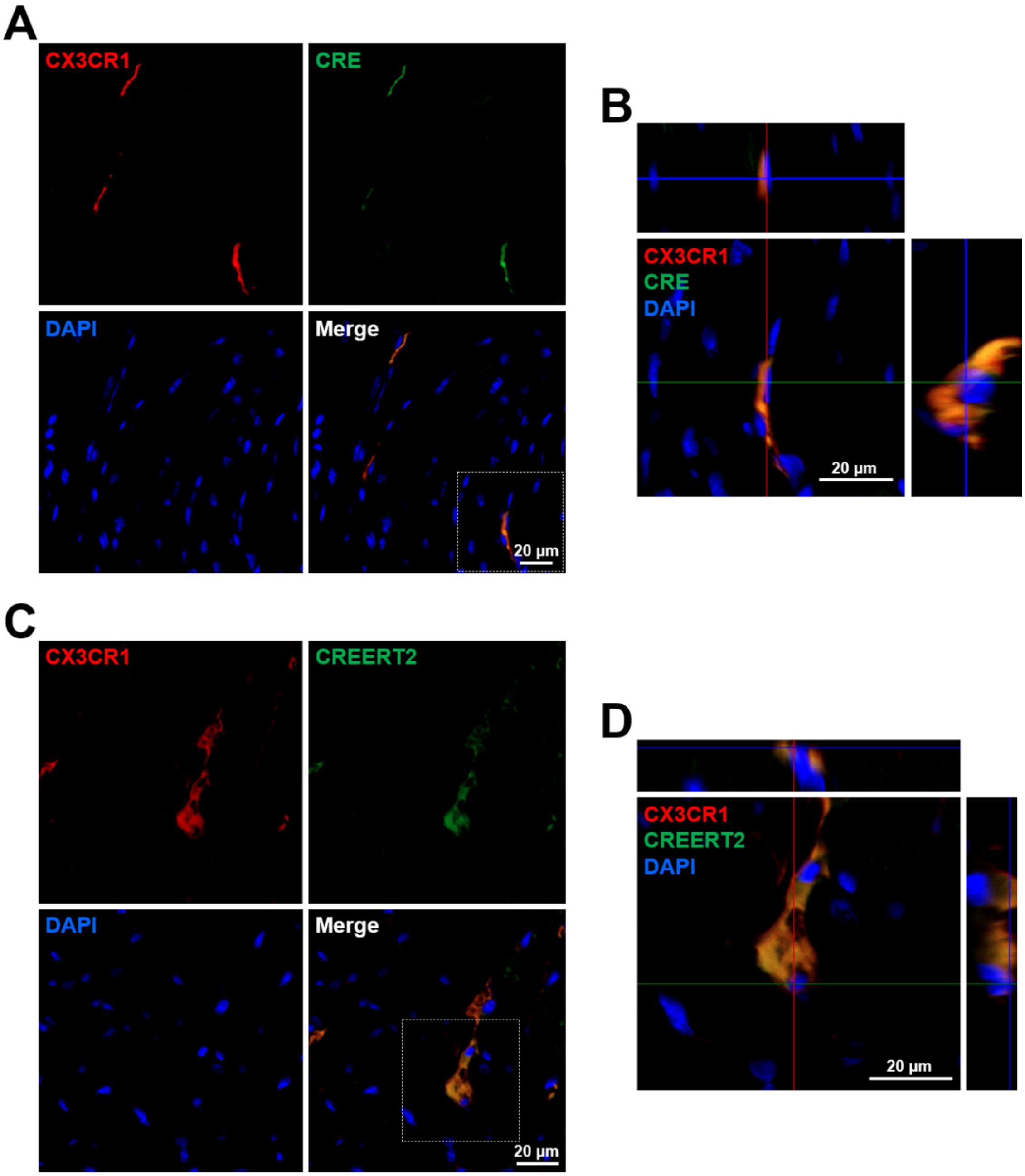

Appendix Figure S3 Assessment of specificity and leakiness of *Cx3cr1-Cre* and *Cx3cr1-CreERT2* mice. A-D. Representative confocal microscopic images of *Cx3cr1-Cre* (A-B) and *Cx3cr1-CreERT2* (C-D) adult (3-month-old) mouse hearts immunostained for CRE and CX3CR1. **B** and **D.** Orthogonal microscopic images of boxed area in panels A and C, respectively. CX3CR1 was visualized with Alexa Fluor 647 and CRE/CREERT2 were visualized with Alexa Fluor 488. DAPI (blue).

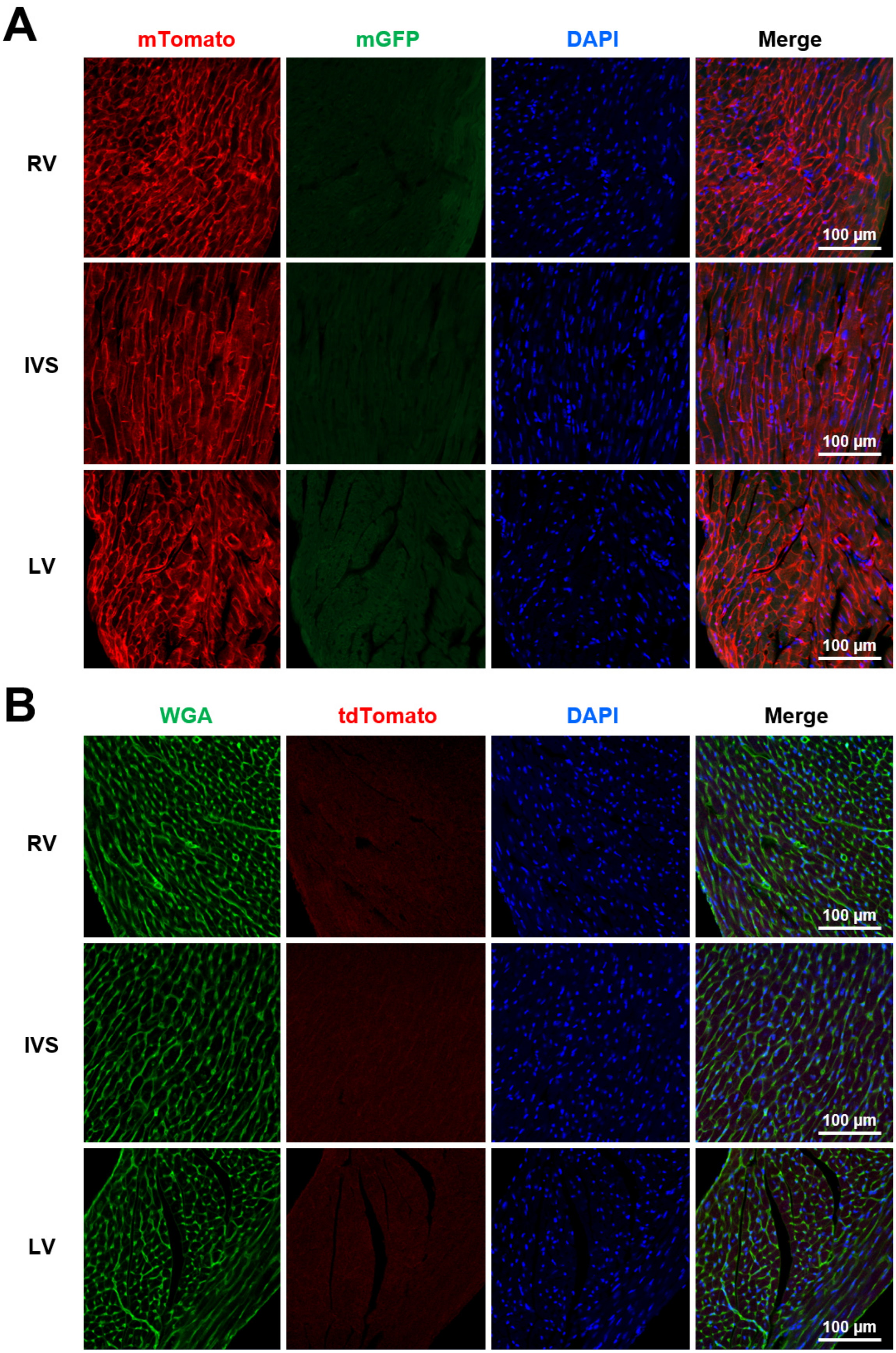
A
mTomato
mGFP
DAPI
Merge
RV
IVS
LV
100 μm
B
WGA
tdTomato
DAPI
Merge
RV
IVS
LV
100 μm

Appendix Figure S4. Assessment of leakiness of *R26-mT/mG* and *R26-tdTomato* reporter mice. A. Representative confocal microscopic images of *R26-mT/mG* adult mouse hearts. mGFP signal was not detected in the whole heart. **B.** Representative confocal microscopic images of *R26-tdTomato* adult mouse hearts. Cellular membrane was visualized with wheat germ agglutinin (WGA). tdTomato signal was not detected in the whole heart. RV, right ventricle; IVS, interventricular septum; LV, left ventricle.

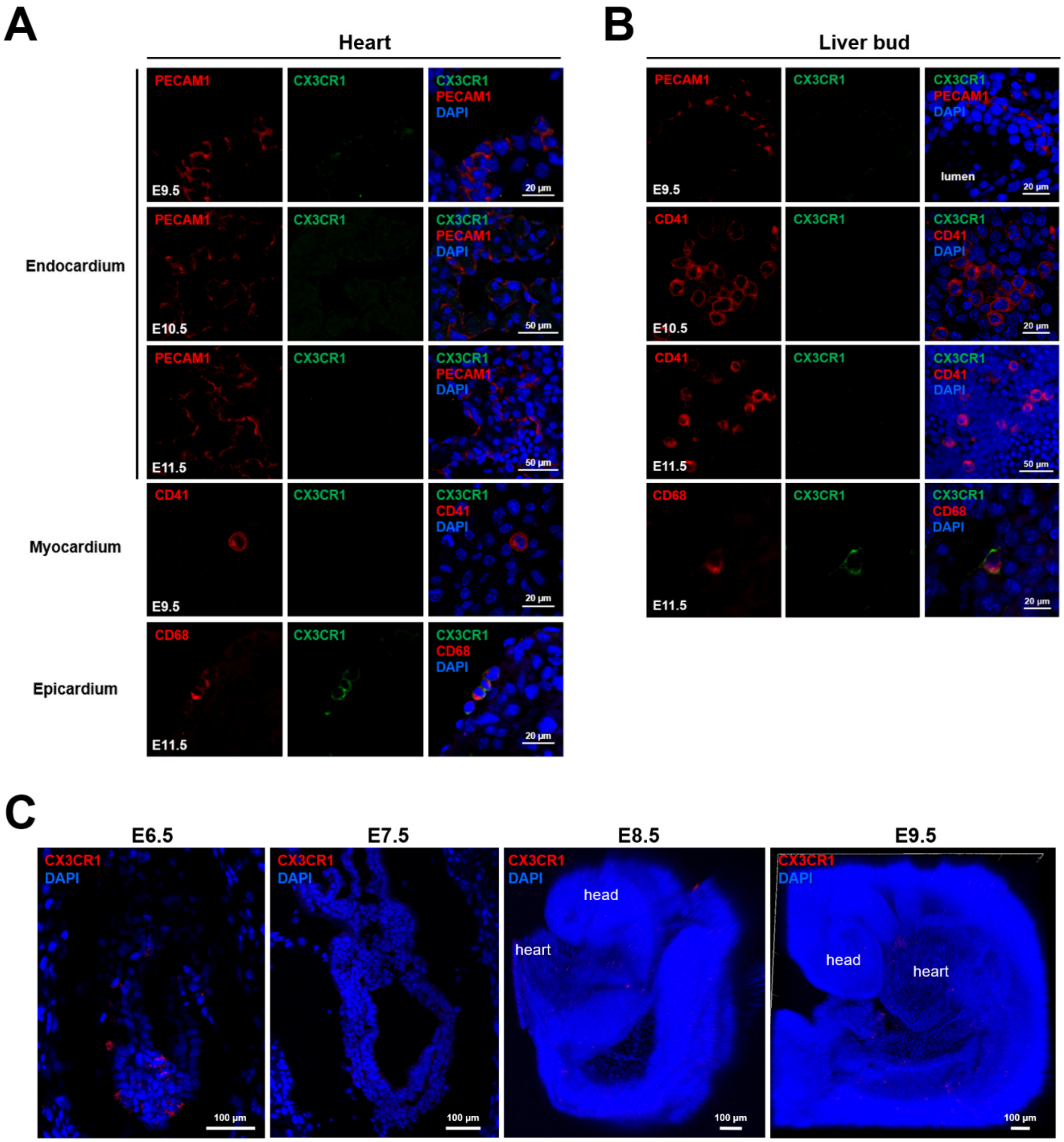

Appendix Figure S5. Expression profiling of CX3CR1 in the developing embryo. A-C. Representative confocal microscopic images of the developing heart (A), liver bud (B), and whole embryos (C) at indicated developmental stages. Mouse embryos were immunostained for CX3CR1, PECAM1, CD41, and CD68. DAPI (blue).

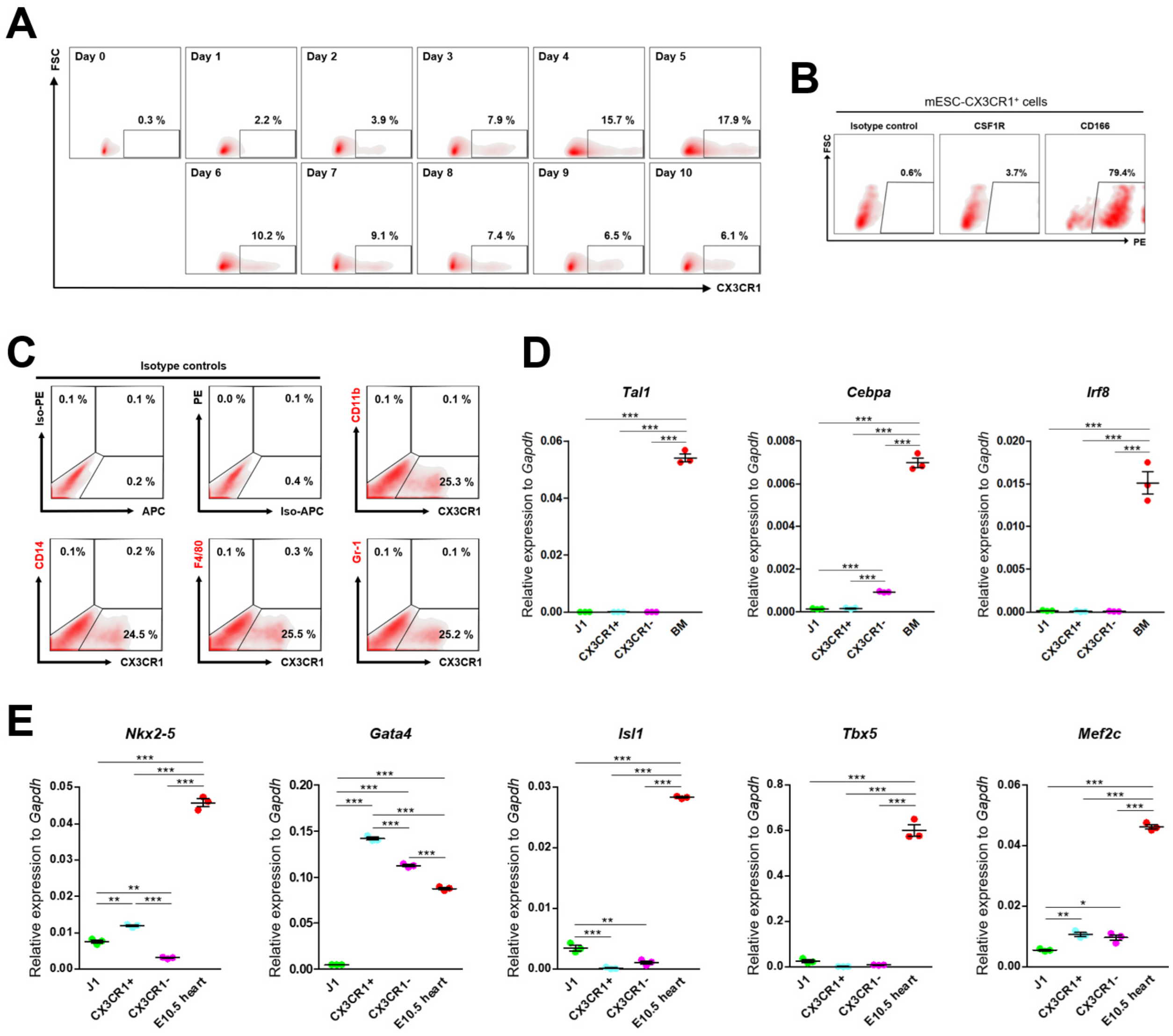

Appendix Figure S6. Characterization of mESC-CX3CR1⁺ cells *in vitro*. A. Flow cytometric analysis for CX3CR1 with differentiating mESCs cultured with OP9 cells from D0 to D10. **B.** Flow cytometric analysis of mESC-CX3CR1⁺ cells. mESC-CX3CR1⁺ cells were further gated for analysis of CSF1R and CD166 expression at D5. **C.** Flow cytometric analysis for myeloid surface markers with differentiating mESCs cultured with OP9 cells at D5. **D-E.** Gene expression profile of myeloid transcription factors (D) and cardiac transcription factors (E) in mESC-CX3CR1⁺ cells. Differentiating mESCs at Day 5 were subjected to MACS using an anti-CX3CR1 antibody. Sorted cells (CX3CR1⁺ cells and CX3CR1⁻ cells) together with J1 mESCs, bone marrow cells (D), and E10.5 mouse hearts (E) were subjected to RNA extraction and qRT-PCR. Error bars: standard

error of mean. One-way ANOVA was performed followed by a Tukey HSD test. *$P < 0.05$, **$P < 0.01$, ***$P < 0.001$. BM, bone marrow.

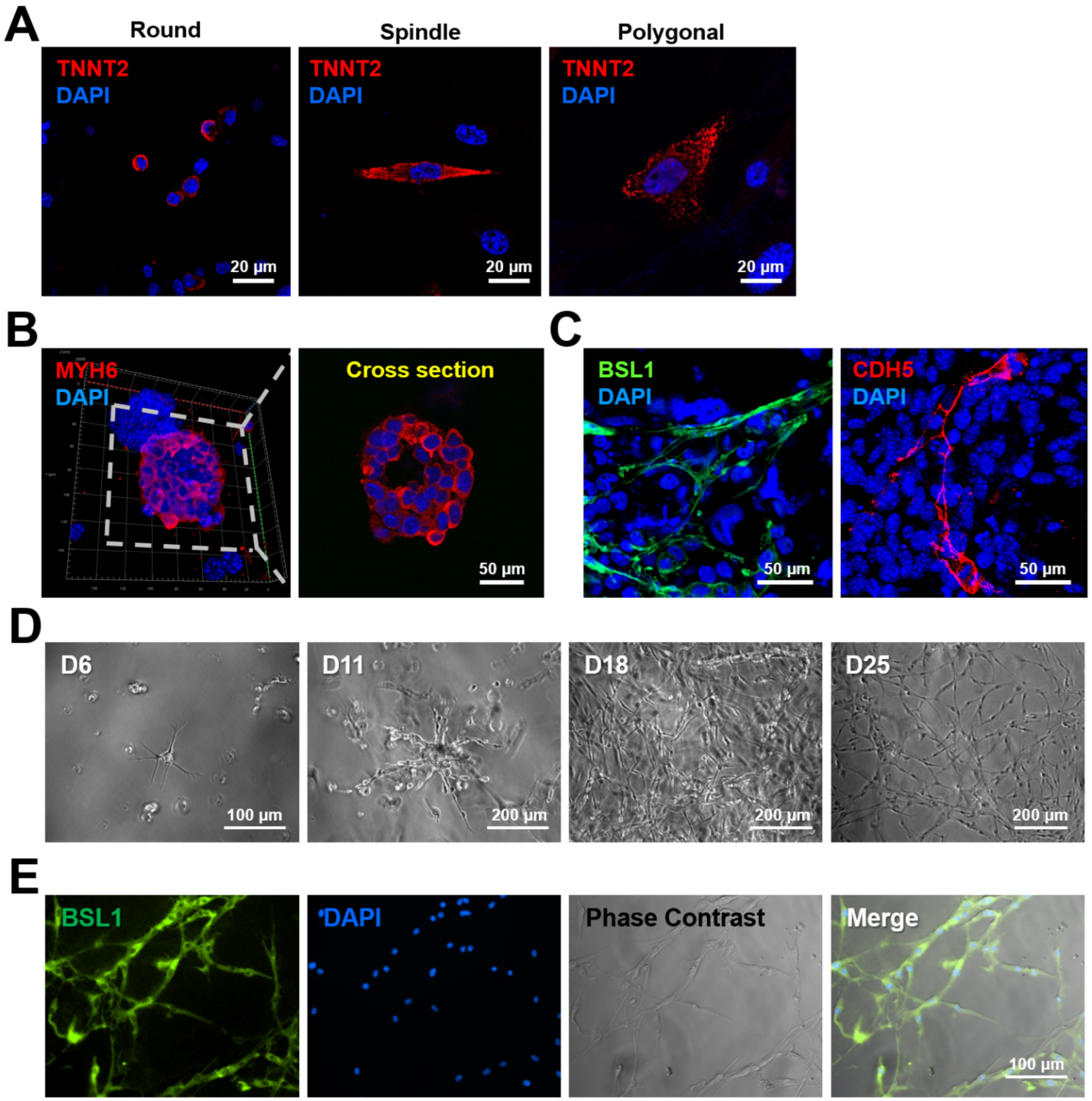

Appendix Figure S7. Differentiation of mESC-CX3CR1^{+} cells into cardiomyocytes and endothelial cells *in vitro*. A-C. Representative confocal microscopic images of cultured mESC-CX3CR1^{+} cells stained for CM markers TNNT2 (A) and MYH6 (B), and EC markers BSL1 and CDH5 (C). MACS-isolated cells were cultured in CM differentiation conditions as shown in Figure 6A (upper). Cells were harvested at D15-20 and subjected to ICC. DAPI (blue). **D.** Representative phase contrast microscopic images of the MACS-isolated mESC-CX3CR1^{+} cells at the indicated time points. MACS-isolated cells were cultured in endothelial growth medium (EGM2) on top of

Matrigel over 25 days as shown in Figure 6A (lower). **E.** Epifluorescence microscopic images of cells stained with BSL1 at D25. DAPI (blue).

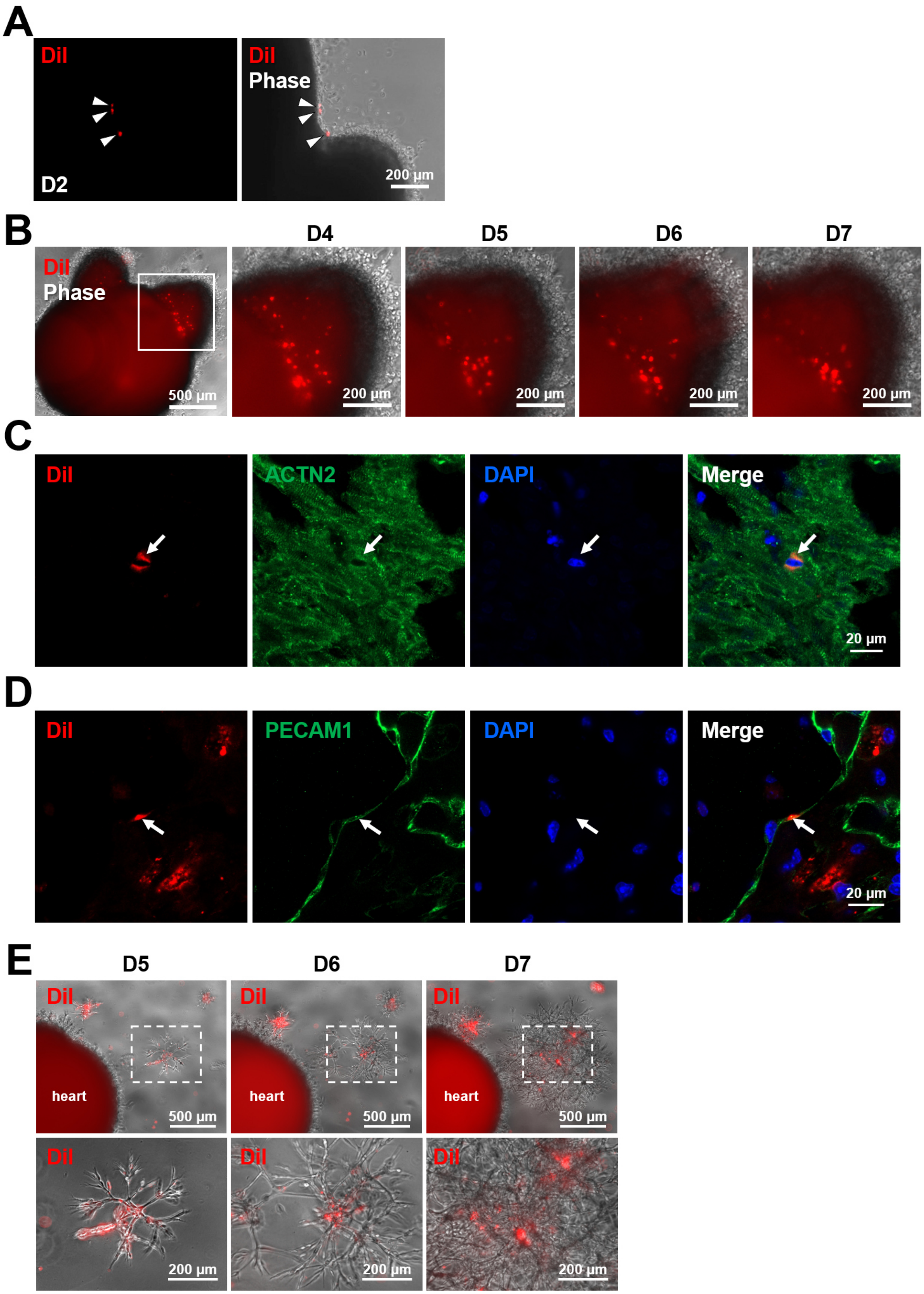
A
DiI
D2
DiI
Phase
200 µm
B
DiI
Phase
500 µm
D4
D5
D6
D7
200 µm
C
DiI
ACTN2
DAPI
Merge
20 µm
D
DiI
PECAM1
DAPI
Merge
20 µm
E
D5
D6
D7
DiI
heart
500 µm
200 µm

Appendix Figure S8. Differentiation of mESC-CX3CR1^{+} cells into cardiomyocytes and endothelial cells in the fetal heart *ex vivo*. FACS-isolated mESC-CX3CR1^{+} cells were labeled with DiI and loaded on top of Matrigel containing E15.5 fetal mouse hearts as shown in Figure 6D. **A.** Representative fluorescent and phase contrast microscopic images of *ex vivo* fetal mouse hearts at D2 (n = 12). Arrow heads indicate DiI-labeled CX3CR1^{+} cells derived from mESCs. **B.** Epifluorescence microscopic images of DiI^{+} cells incorporated into the fetal mouse hearts at D4-7. **C-D.** Representative confocal microscopic images of fetal mouse hearts stained for ACTN2 (C) and PECAM1 (D). Hearts were harvested at D20 and subjected to IHC. Arrows indicate CMs (C) and ECs (D) contributed by mESC-CX3CR1^{+} cells. DAPI (blue). **E.** Epifluorescence and phase contrast images of mESC-CX3CR1^{+} cells sprouting and forming networks outside of the fetal mouse heart within the Matrigel at D5-7. Boxed areas were magnified in lower images.

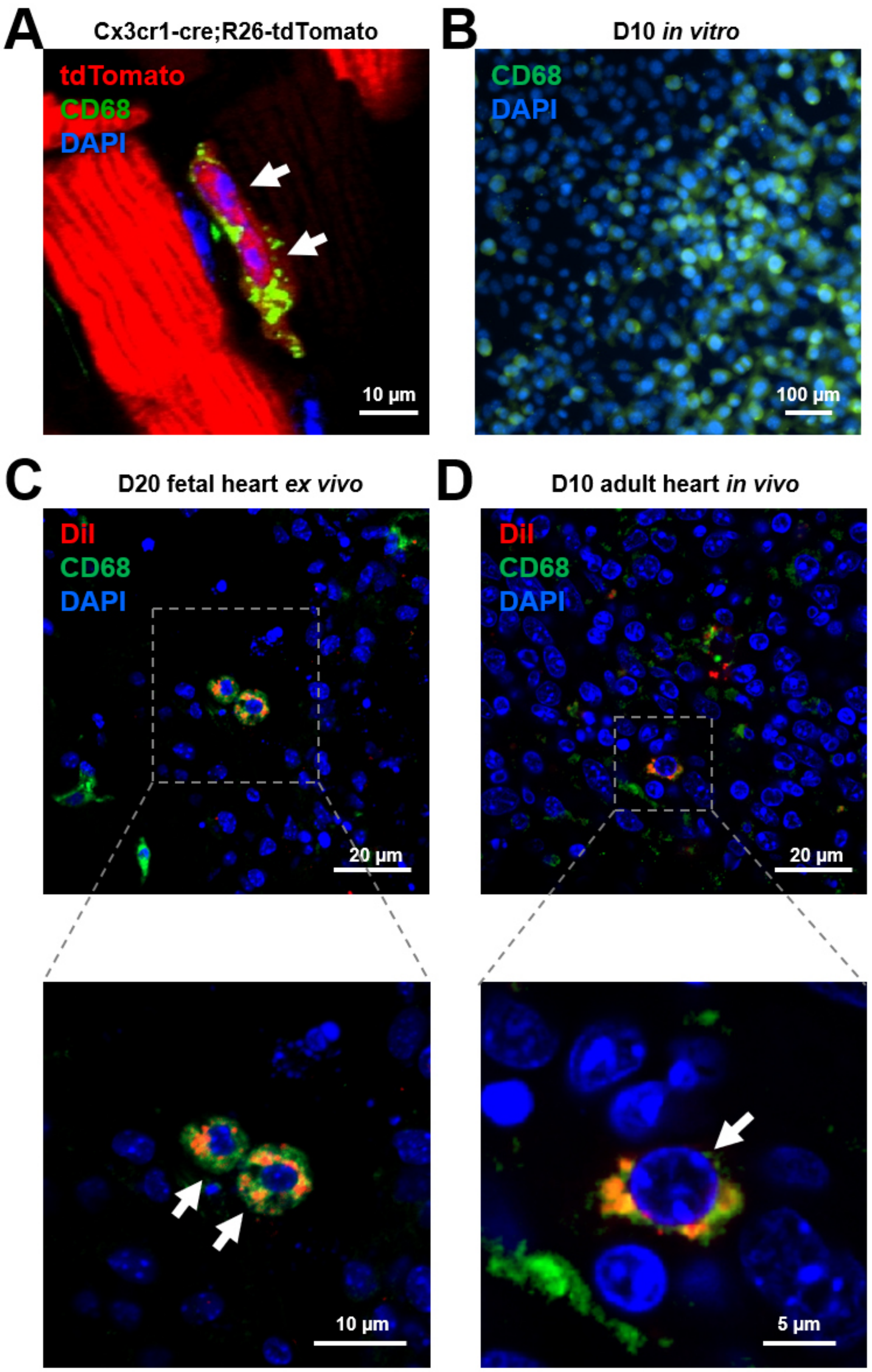

Appendix Figure S9. Contribution of CX3CR1$^+$ cells to cardiac macrophages *in vivo*, *in vitro*, and *ex vivo*. A. A representative confocal microscopic image of the adult (6-month-old) heart of *Cx3cr1-cre;R26-tdTomato* mice. Heart tissues were sectioned and stained for CD68 (green). tdTomato (red). DAPI (blue). **B.** A representative epifluorescence microscopic image of cultured

mESC-CX3CR1$^+$ cells stained for CD68 at D10**.** MACS-isolated mESC-CX3CR1$^+$ cells at D5 were cultured in the presence of M-CSF for an additional 5 days. **C.** Representative confocal microscopic images of fetal mouse hearts stained for CD68 at D20. FACS-isolated mESC-CX3CR1$^+$ cells were labeled with DiI and loaded on top of Matrigel containing E15.5 fetal mouse hearts as shown in Figure 6D. DAPI (blue). A boxed area in the upper panel was magnified in the below panel. **D.** Representative confocal microscopic images of adult mouse hearts stained for CD68 at D10. FACS-isolated mESC-CX3CR1$^+$ cells were labeled with DiI, encapsulated within PA-RGDS, and injected into the adult mouse hearts as shown in Figure 6G. DAPI (blue). A boxed area in the upper panel was magnified in the below panel.

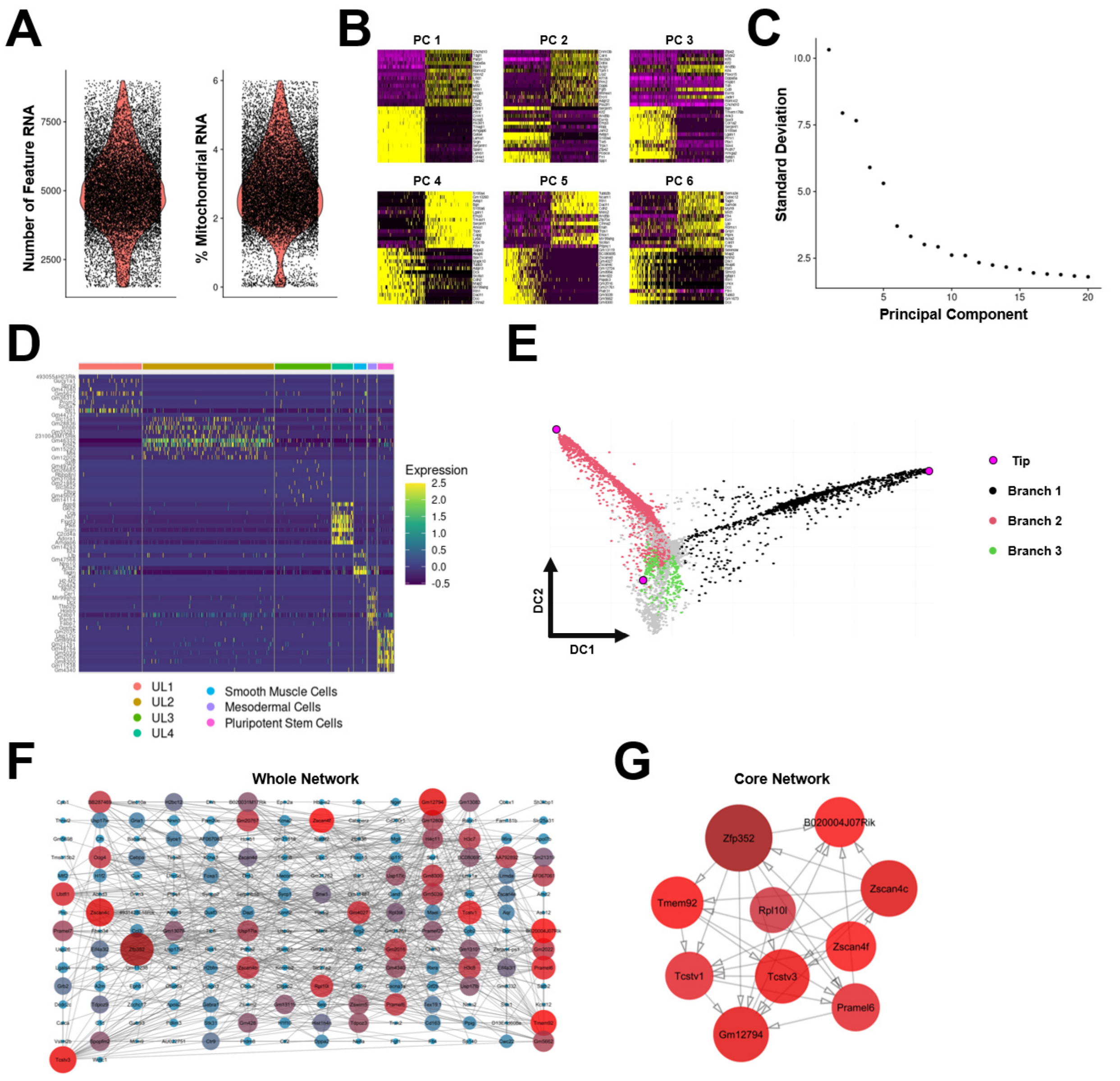

H

Gene	Full name	Function	PMID
Zfp352	Zinc finger protein 352	Activation of developmental programs during embryogenesis	37778747
Zscan4	Zinc finger and SCAN domain containing 4	Derepression of heterochromatin in mESCs	26324425
		Maintenance of telomere length and genome stability in mESCs	20336070
Tcstv	2 cell stage variable group member	Maintenance of telomere length in mESCs	26816107

Appendix Figure S10. scRNA-seq analysis of differentiating mESCs cultured with OP9 cells at D5. J1 mESCs and OP9 cells were cocultured for 5 days and subjected to scRNA-seq as shown in Figure 7A. **A.** Filtering out low-quality cells. Cells with a total number of feature RNA between 1,500 and 9,000 were included for further analysis (left). In addition, cells with more than

6% of mitochondrial genes were excluded from further analysis (right). **B.** Principal component analysis (PCA). **C.** Jackstraw permutation test. **D.** A heatmap for differentially expressed genes (DEGs) across all clusters. The top 10 differentially expressed genes (DEGs) were identified using a significance threshold set at log2FC (Fold Change) ≥ 0.25 and P value ≤ 0.05. **E.** A diffusion map showing three major differentiation trajectories. **F.** The whole protein-protein interaction (PPI) network of *Cx3cr1*$^{+}$ cells in UL4 cluster. The PPI was retrieved using the STRING database and was subsequently analyzed by Cytoscape. The node size represents the interaction degree of each gene while the color coding indicates their expression levels in scRNA-seq analysis with red denoting upregulated genes and blue indicating downregulated genes. **G.** The core network derived from the whole network in panel F. **H.** A table summarizing the function of key transcription factors in the core network in panel G.

Appendix Table S1. Materials used for IHC and ICC

Material	Manufacturer	Catalog #
anti-Laminin	Sigma Aldrich	L9393
anti-TNNT2	Abcam	ab8295
anti-ACTN2	Abcam	ab5694
anti-PECAM1	BD	550274
anti-CD68	AbD Serotec	MCA19575
anti-KDR	Cell Signaling Technologies	2479
anti-CX3CR1	BioLegend	149007
anti-CD45	BD	559864
anti-CRE	Cell Signaling Technologies	15036T
anti-CD41	BioLegend	133901
anti-MYH6	Abcam	ab15
anti-CDH5	BD	550548
BSL1	Vector Laboratories	FL-1101
CM-Dil Dye	Thermo Fisher	C7001

Appendix Table S2. Materials used for flow cytometry and MACS

Material	Manufacturer	Catalog #
anti-TNNT2	Abcam	Ab8295
anti-CX3CR1	BioLegend	149007
anti-CSF1R	Miltenyi Biotec	130-102-962
anti-CD166	Miltenyi Biotec	130-105-444
anti-CD11b	BD	557397
anti-CD14	BD	553740
anti-F4/80	BioLegend	123115
anti-Gr-1	BD	553128
BSL1	Vector Laboratories	FL-1101
anti-APC magnetic beads	Miltenyi Biotec	120-001-265

Appendix Table S3. Primer sequences used for qRT-PCR analysis

Target gene	Forward sequence	Reverse sequence
Tal1	CACTAGGCAGTGGGTTCTTTG	GGTGTGAGGACCATCAGAAATCT
Cebpa	CAAGAACAGCAACGAGTACCG	GTCACTGGTCAACTCCAGCAC
Irf8	CGGGGCTGATCTGGGAAAAT	CACAGCGTAACCTCGTCTTC
Nkx2-5	GACAAAGCCGAGACGGATGG	CTGTCGCTTGCACTTGTAGC
Gata4	CCCTACCCAGCCTACATGG	ACATATCGAGATTGGGGTGTCT
Isl1	ATGATGGTGGTTTACAGGCTAAC	TCGATGCTACTTCACTGCCAG
Tbx5	ATGGCCGATACAGATGAGGG	TTCGTGGAACTTCAGCCACAG
Mef2c	ATCCCGATGCAGACGATTCAG	AACAGCACACAATCTTTGCCT
Gapdh	ATGACCACAGTCCATGCCATC	CCTGCTTCACCACCTTCTTG
