# Supplementary figures and images for: Epiblast-derived CX3CR1+ progenitors generate cardiovascular cells during cardiogenesis
Source: EMBO J. 2025 Jun 23;44(15):4331–51. doi: 10.1038/s44318-025-00488-z (PMC12316875; doi:10.1038/s44318-025-00488-z)

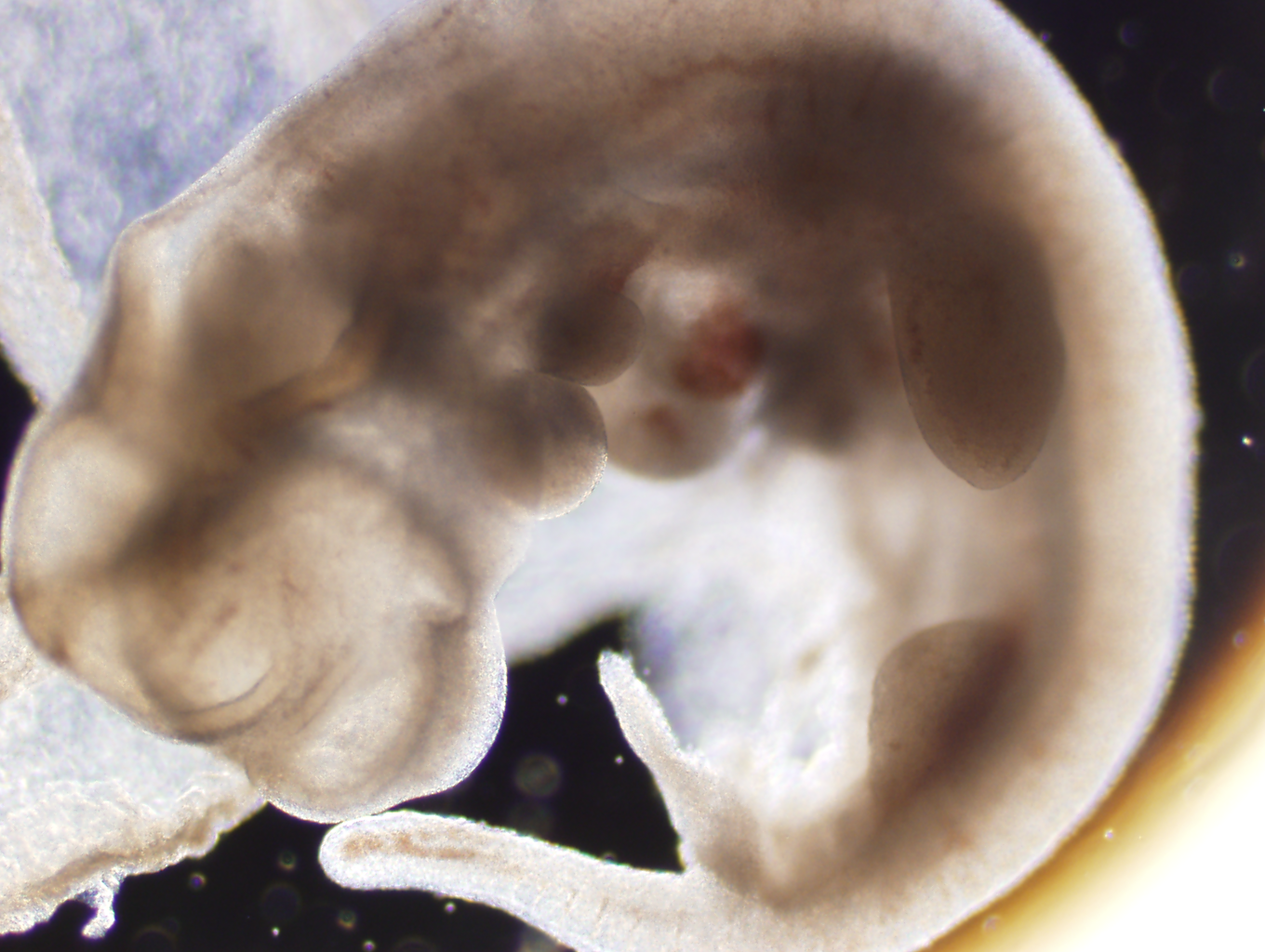

Supplement: Supplementary file 7 — Source data Fig. 5 [file 44318_2025_488_MOESM7_ESM.zip › Figure 5/5C-left.tif]

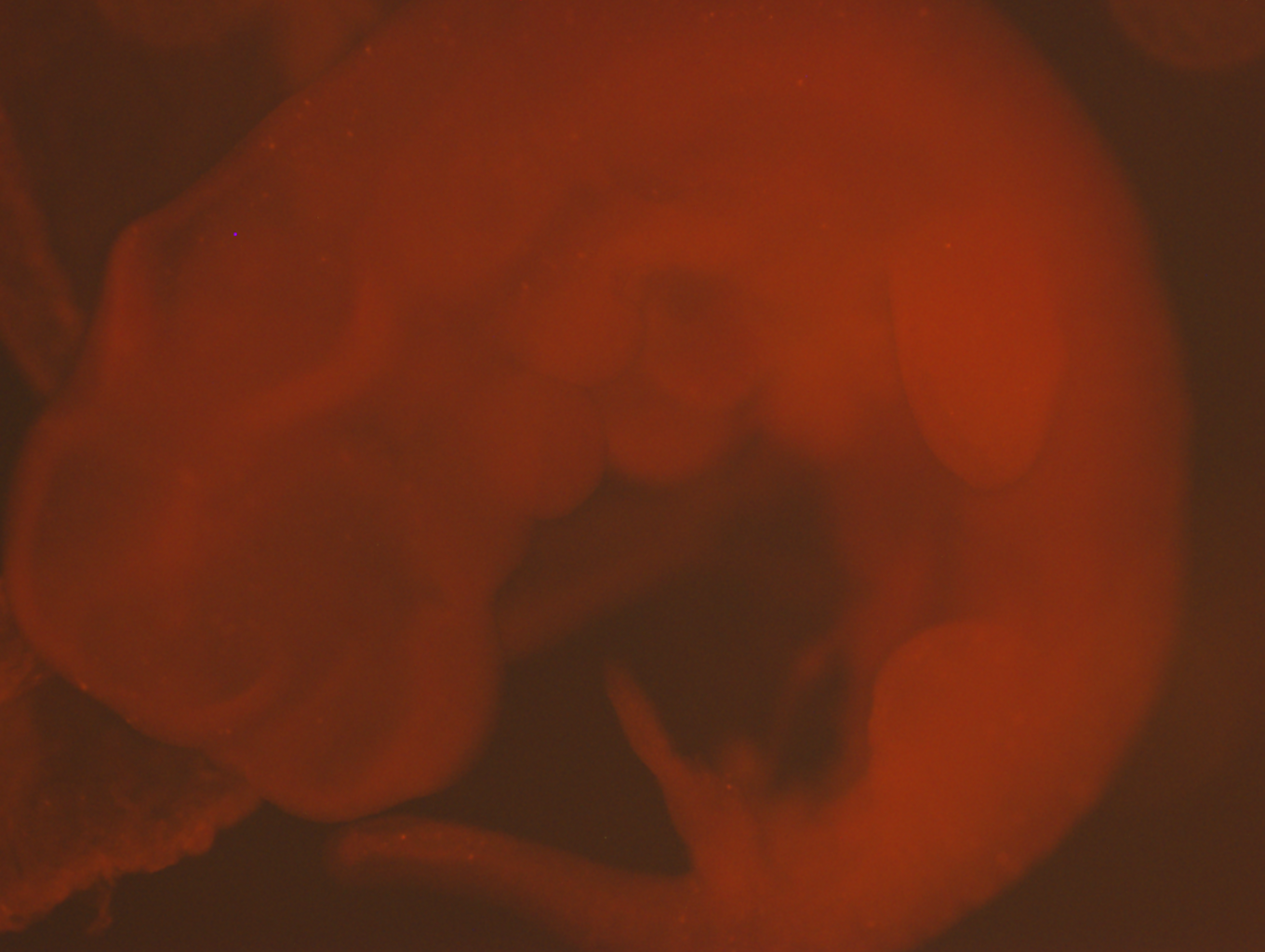

Supplement: Supplementary file 7 — Source data Fig. 5 [file 44318_2025_488_MOESM7_ESM.zip › Figure 5/5C-right.tif]

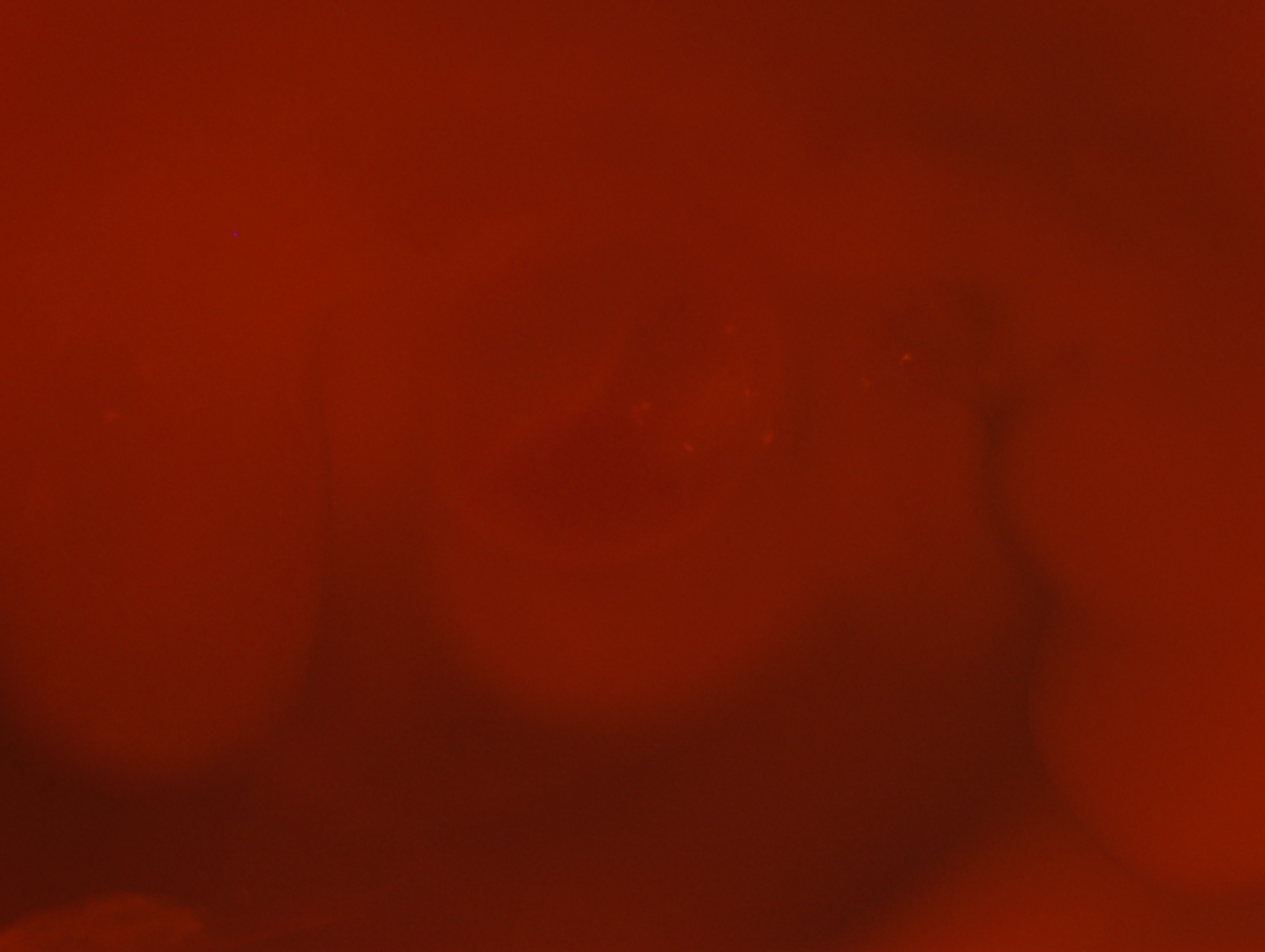

Supplement: Supplementary file 7 — Source data Fig. 5 [file 44318_2025_488_MOESM7_ESM.zip › Figure 5/5D-left.tif]

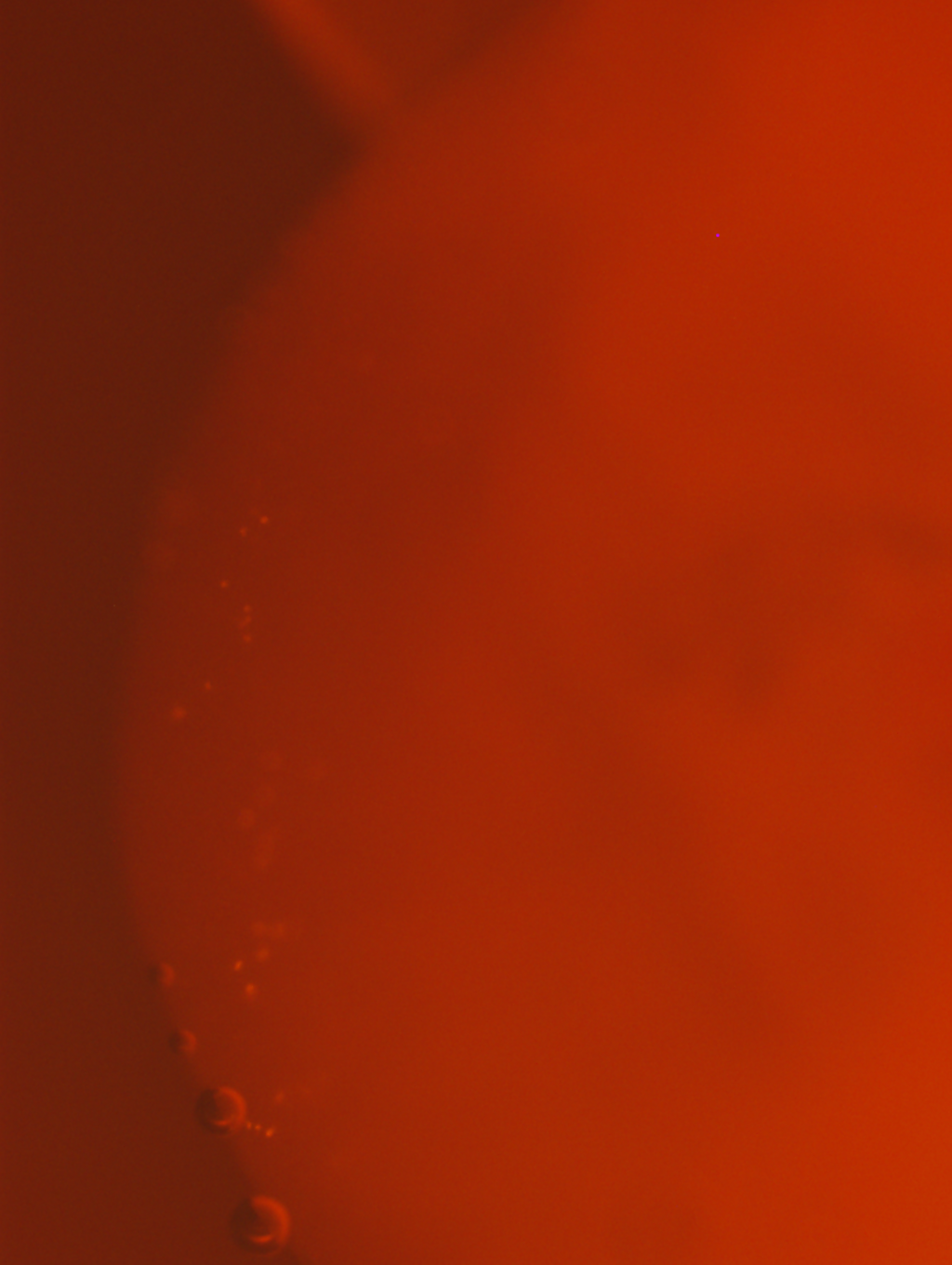

Supplement: Supplementary file 7 — Source data Fig. 5 [file 44318_2025_488_MOESM7_ESM.zip › Figure 5/5D-right.tif]

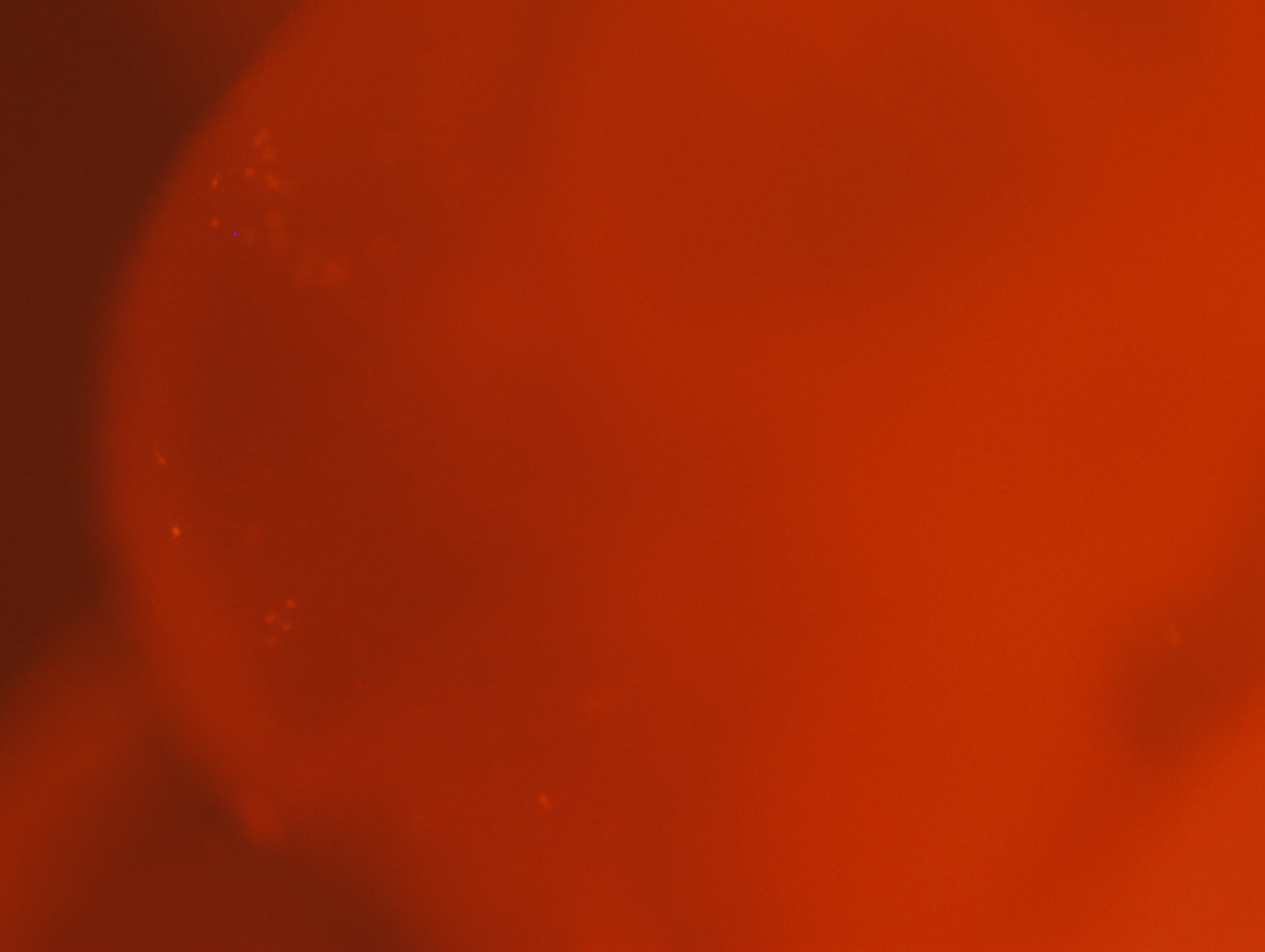

Supplement: Supplementary file 7 — Source data Fig. 5 [file 44318_2025_488_MOESM7_ESM.zip › Figure 5/5D-middle.tif]
